# Anatomy of Permanent‎ ‎Mandibular‎ First‎ Molars in a Selected Iranian Population Using ‎Cone-beam Computed Tomography 

**DOI:** 10.22037/iej.v13i2.19035

**Published:** 2018

**Authors:** Aria Choupani Dastgerdi, Manije Navabi, Ladan Hafezi, Zohre Khalilak, Vahid Rakhshan

**Affiliations:** a *Private Practice, Tehran, Iran; *; b *Department of Removable Prosthodontics, Dental Branch, Islamic Azad University, Tehran, Iran; *; c *Department of Oral Radiology, Dental Branch, Islamic Azad University, Tehran, Iran; *; d *Department of Endodontics, Dental Branch, Islamic Azad University, Tehran, Iran*

**Keywords:** Anatomy, Cone-beam Computed Tomography, Endodontics, ‎ Root Anatomy

## Abstract

**Introduction:**

Knowledge of radicular anatomy has a crucial impact on endodontic practices. Since some anatomic features such as modifications of Vertucci are not evaluated adequately, this study was conducted.

**Methods and Materials:**

In this *in vivo* study, cone-beam computed tomography (CBCT) images of‎ 312‎ intact bilateral first‎ molars‎ from‎ 156‎ patients‎ (‎79‎ ‎males‎ and‎ 77‎ females with an average age of ‎35.58‎±‎11.17‎ years‎)‎ were‎ investigated by a trained dentist in terms of number‎ of‎ roots,‎ number‎ of‎ canals‎ in‎ each‎ root‎ and‎ in‎ ‎each‎ tooth,‎ and shapes‎ of‎ canals‎ according‎ to‎ Vertucci’s‎ classification‎ and‎ its‎ modifications.‎ Groups were compared using the *Chi*-square test. The level of significance was set at 0.05.

**Results:**

Of all teeth, 5.2%‎ had‎ 3‎ roots.‎‎ ‎Mesial‎ roots‎ had‎ mostly‎ 2‎ canals‎ while distal roots had a similar frequency of 1 and 2 canals.‎ ‎Of‎ all‎ teeth,‎ ‎‎39.7%‎ had‎ 3‎ canals,‎ 45.2%‎ had‎ 4‎ canals,‎ 13.8%‎ had‎ 5‎ canals,‎ and‎ 1.3%‎ had‎ 6‎ canals.‎ There were no significant differences between males and females, ‎in terms of number of roots (*P*=0.137), number of canals in mesial (*P*=0.453) or distal roots (*P*‎‎=0.328), and total number of canals (*P*=0.138).‎ The most frequent Vertucci classes in mesial and distal roots were IV ‎‎followed‎ by‎ II and I‎, respectively. There were no significant differences between males and females in terms of Vertucci classes of mesial (*P*=0.211) or distal (*P*=0.205) roots.

**Conclusion:**

In this population, there were 3 to 6 canals per tooth (mostly 4 and 3 canals).‎ Males and female’s ‎might be similar regarding the number of roots, or number of canals in each root, number of ‎canals in each tooth, or the predominant canal shape in each root.

## Introduction

Proper‎ knowledge‎ of‎ the‎ internal‎ anatomy‎ of‎ root‎ canals‎ is‎ crucial‎ for‎ a‎ successful‎ endodontic‎ treatment‎ which‎ relies‎ on‎ appropriate‎ cleaning‎ and‎ shaping‎ [1, 2].‎ Since‎ mandibular‎ first‎ molars‎ emerge‎ very‎ early‎ in‎ the‎ mouth‎ and‎ have‎ complex‎ surfaces,‎ they‎ are‎ one‎ of‎ the‎ teeth‎ most‎ requiring‎ root‎ canal‎ therapy;‎ and‎ therefore‎ the‎ knowledge‎ of‎ their‎ root‎ canal‎ anatomy‎ is‎ important‎ [2, 3].‎ These‎ teeth‎ usually‎ have‎ three‎ canals‎ within‎ two‎ roots‎ although‎ they‎ can‎ change‎ due‎ to‎ ethnicity‎ or‎ normal‎ variations‎ [4-7].‎ ‎ 

Since‎ ethnic‎ background‎ can‎ affect‎ root‎ anatomy‎ of‎ mandibular‎ first‎ molars,‎ it‎ is‎ important‎ to‎ document‎ properties‎ of‎ these‎ teeth‎ in‎ various‎ populations.‎ However,‎ studies‎ on‎ Iranians‎ are‎‎ few and controversial [2, 3, 8-11].‎ Many‎ aspects‎ of‎ anatomic‎ features‎ are‎ not‎ usually covered‎ by‎ them (such as modifications of Vertucci classification which is not covered in many studies worldwide).‎ Many of older‎ studies‎ have‎ used‎ conventional‎ or‎ 2D‎ radiography‎ techniques‎ that‎ can be‎ less‎ accurate‎ than‎ 3D‎ radiography techniques [12]. Nevertheless, recent studies have mainly used cone‎-beam‎ computed‎ tomography‎ (CBCT) due to its numerous advantages [8-11].‎ 

This study evaluated number and shape of roots and canals of permanent mandibular first molars using CBCT images of an Iranian sample population.

**Figure 1:**
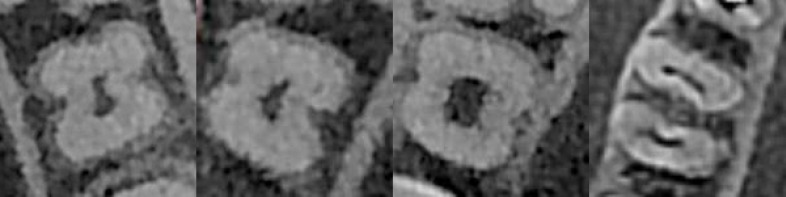
Examples of transverse CBCT sections

**Figure 2:**
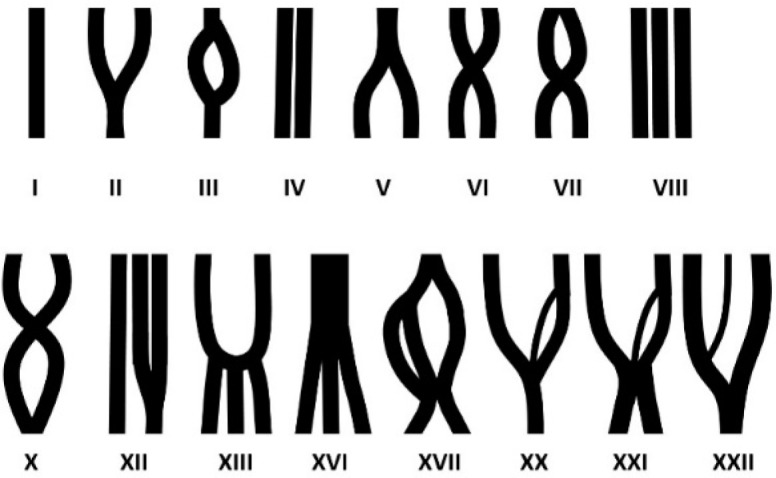
Schematic diagrams representing canals within a root [black columns drawn together from top (coronal) to bottom (apical)], arranged as all Vertucci classes (top row) and as certain Vertucci modifications that were observed in this sample of mandibular first molars (bottom row)

## Materials and Methods

This‎ *in‎ vivo*‎ study‎ was‎ performed‎ on‎ CBCT‎ images‎ of‎ patients‎ aged‎ 20‎ to‎ 60‎ years‎‎ who‎ had‎ attended‎ two‎ centers in Tehran‎.‎ All‎ CBCTs‎ had‎ been‎ retrospectively‎ taken‎ solely‎ for‎ clinical‎ purposes.‎ No‎‎ x-ray‎ was‎ emitted‎ to‎ patients‎ for‎ this‎ study,‎ and‎ study‎ ethics‎ were‎ approved‎ by‎ the research‎ committee‎ of‎ Azad University of Medical Sciences, Dental branch, Tehran, Iran (#14859).‎ All‎ CBCTs‎ had‎ been‎ taken‎ with‎ the‎ same‎ unit‎ (NewTom,‎ GiANO,‎ Verona,‎ Italy);‎ with similar field‎ of‎ view‎ (8×5 cm),‎ focal‎ size‎ (0.3‎ mm),‎ current (12 mA),‎ peak kilovoltage‎ (85 kVp), and time (0.4‎ sec).‎ Inclusion‎ criteria‎ were‎ availability‎ of‎ both‎ mandibular‎ first‎ molars‎ and in‎ each‎ patient,‎ full‎ patient‎ information was obtained. Exclusion criteria were‎ resorption, ‎previous‎ endodontic‎ treatment,‎ open‎ apex,‎ agenesis,‎ fractures,‎ or‎ pathologies.‎ A‎ total‎ of‎ 312‎ images of first‎ molars‎ from‎ 156‎ patients‎ were‎ included.‎ 

All‎ measurements‎ were‎ done‎ by‎ the‎ same‎ dentist‎ trained‎ by‎ a‎ maxillofacial‎ radiologist,‎ using‎ NTT Viewer software program (NTT Software Corporation, Yokohama, Japan).‎ CBCT‎ images‎ were‎ examined‎ in‎ coronal,‎ sagittal,‎ and‎ mainly‎ axial‎ dimensions (Figure 1).‎ Evaluated‎ parameters‎ were‎ number‎ of‎ roots,‎ number‎ of‎ canals‎ in‎ each‎ root‎ and‎ in‎ each‎ tooth,‎ and shapes‎ of‎ canals‎ according‎ to‎ the Vertucci‎ classification‎ [13]‎ and‎ its‎ modifications‎ [14-17].‎ Vertucci‎ classes‎ can‎ be‎ summarized‎ based‎ on‎ the‎ number‎ of‎ canals‎ from‎ coronal‎ to‎ apical‎ portions‎ of‎ the‎ root:‎ type‎ I‎ (coronal‎ canals:‎ 1,‎ apical‎ canals:1‎ [1-1]),‎ type‎ II‎ (2-1),‎ type‎ III‎ (1-2-1),‎ type‎ IV‎ (2-2),‎ type‎ V‎ (1-2),‎ type‎ VI‎ (2-1-2),‎ type‎ VII‎ (1-2-1-2),‎ type‎ VIII‎ (3-3),‎ type‎ IX‎ (3-1),‎ type‎ X‎ (3-1-2-1),‎ type‎ XI‎ (4-2),‎ type‎ XII‎ (3-2),‎ type‎ XIII‎ (2-3),‎ type‎ XIV‎ (4-4),‎ type‎ XV‎ (5-4),‎ type‎ XVI‎ (1-3),‎ type‎ XVII‎ (1-2-3-2),‎ type‎ XVIII‎ (1-2-3),‎ type‎ XIV‎ (3-1-2),‎ type‎ XX‎ (2-3-1),‎ type‎ XXI‎ (2-3-2),‎ type‎ XXII‎ (3-2-1),‎ and‎ type‎ XXIII‎ (3-2-3). Schematic diagrams of Vertucci classes and certain modifications observed are presented in Figure 2.

Descriptive‎ statistics‎ were‎ calculated. Groups were compared using the *chi*-square of Statistical Package for Social Science (SPSS, version 24.0, SPSS, Chicago, IL, USA)‎.‎ The level‎ of‎ significance‎ was predetermined as 0.05.

## Results

The observer was calibrated through repeating the diagnosis of cases (especially more difficult cases) under the supervision of a dental anatomist and an endodontist. Of‎ 156‎ enrolled‎ patients,‎ 79‎ were‎ males‎ and‎ 77‎ were‎ females.‎ Patients’‎ average‎ age‎ was‎ 35.58‎±‎11.17‎ years.‎ Of‎ patients,‎ 101‎ (64.7%)‎ were aged‎ between‎ 20‎ and‎ 39‎ years‎ old,‎ while‎ 55‎ (35.3%) were‎ between‎ 40‎ and‎ 60‎ years‎ old.

***Number*** ***‎*** ***of*** ***‎*** ***roots*** ***‎*** ***and*** ***‎*** ***canals***

Table‎ 1‎ summarizes‎ the number‎ of‎ canals.‎ Among‎ 312‎ assessed‎ teeth,‎ 16‎ (5.2%)‎ bilateral‎ teeth‎ in‎ 8‎ patients‎ had‎ 3‎ roots;‎ in‎ all‎ these‎ cases,‎ the‎ third‎ root‎ was‎ distolingual.‎ All‎ other‎ teeth‎ had‎ 2‎ roots.‎ Mesial‎ roots‎ had‎ mostly‎ 2‎ canals‎ and‎ in‎ few‎ cases‎ 3‎ canals‎ (Table‎ 1).‎ Distal‎ roots‎ showed‎ a‎ rather‎ similar‎ distribution‎ of‎ 1‎ and‎ 2‎ canals,‎ in‎ addition‎ to‎ very‎ few‎ 3‎ canals‎ (Table‎ 1).‎ All‎ distolingual‎ roots‎ contained‎ only‎ 1‎ canal.‎ Overall,‎ number‎ of‎ canals‎ ranged‎ between‎ 3‎ and‎ 6.‎ Of‎ 312‎ teeth,‎ 39.7%‎ had‎ 3‎ canals,‎ 45.2%‎ had‎ 4‎ canals,‎ 13.8%‎ had‎ 5‎ canals,‎ and‎ 1.3%‎ had‎ 6‎ canals.‎ All‎ 6-canaled‎ teeth‎ were‎ 3-rooted.‎ There were no significant differences between males and females, in terms of number of roots (*P*=0.137), number of canals in mesial (*P*=0.453) or distal roots (*P*=0.328), and total number of canals (*P*=0.138).

***Vertucci*** ***‎*** ***classifications***

Tables‎ 3‎ and‎ 4‎ present‎ Vertucci‎ classes‎ and‎ Vertucci‎ modifications.‎ In‎ mesial‎ roots,‎ the‎ most‎ common‎ classes‎ were‎ type‎ IV‎ followed‎ by‎ II.‎ In‎ distal‎ roots,‎ the‎ most‎ common‎ class‎ was‎ type‎ I‎ (Tables‎ 2‎ and‎ 3, Figure 2).‎ The *Chi*-square did not show significant differences between males and females in terms of Vertucci classes in the mesial root (*P*=0.211) or distal root (*P*=0.205).

## Discussion

The‎ findings‎ of‎ the present‎ study‎ showed‎ that‎ only‎ 5.2%‎ of‎ first‎ molars‎ had‎ three‎ roots.‎ Our‎ results‎ were‎ similar‎ to‎ other‎ Iranian‎ studies‎ reporting‎ prevalence‎ of‎ third‎ root‎s ranging‎ between‎ zero‎ and‎ 3.9%‎ [2, 8-11].‎ Also‎ our‎ findings‎ were‎ consistent‎ with‎ studies‎ on‎ Jordan‎ [14],‎ India‎ [18],‎ Turkey‎ [19, 20]‎ and‎ Sudan‎ [21].‎ However,‎ they‎ were‎ not‎ in‎ line‎ with‎ results‎ from‎ South‎ Korea‎ [22]‎ and‎ Burma‎ [7].‎ It‎ seems‎ that‎ presence‎ of‎ distolingual‎ roots‎ might‎ depend‎ on‎ ethnicity, and results‎ in‎ Asians‎ indicate‎ a‎ rather‎ high‎ prevalence‎ of‎ the‎ third‎ root‎ (Table‎ 4)‎ [4-7, 22, 23].

In‎ this‎ study,‎ prevalence‎ of‎ three‎ and‎ four‎ canals‎ in‎ each‎ tooth‎ were‎ rather‎ similar.‎ This‎ finding‎ was‎ comparable‎ to‎ results‎ of‎ Kim‎ *et‎ al*‎. [22],‎ Zhang‎ *et‎ al*‎.‎ [23],‎ Al-Qudah‎ and‎ Awadeh‎ [14]‎ who‎ reported‎ similar‎ prevalence‎ of‎ three‎ and‎ four‎ canals,‎ but was‎ in‎ contrast‎ to‎ observations‎ of‎ Demirbuga‎ *et‎ al*‎. [20], Chourasia‎ *et‎ al*‎. [18],‎ Ballullaya‎ *et‎ al*‎. [6],‎ Nur‎ [19] and‎ Masoudi‎ [9]‎ who‎ reported‎ a‎ considerably‎ greater‎ prevalence‎ of‎ 3-canaled‎ roots‎ compared‎ to‎ 4-canaled‎ roots. Three-canaled‎ roots‎ can‎ be‎ problematic‎ in‎ endodontic‎ treatments.‎ This‎ research‎ showed‎ prevalence‎ of‎ 3-canaled‎ roots‎ about‎ 13%‎ in‎ mesial‎ roots‎ and‎ 3.5%‎ in‎ distal‎ roots;‎ this‎ was‎ consistent‎ with‎ findings‎ of‎ Al-Qudah‎ and‎ Awawdeh‎ [14],‎ De‎ Pablo‎ *et‎ al*‎. [5]‎ regarding‎ mesial‎ roots‎ and‎ Ahmed‎ *et‎ al*‎. [21]‎ regarding‎ distal‎ root.‎ However,‎ our‎ result‎ was‎ not‎ in‎ line‎ with‎ other‎ reports,‎ which‎ might‎ be‎ due‎ to‎ differences‎ in‎ ethnicity‎ or‎ methodologies.‎ In‎ this‎ study,‎ most‎ of‎ mesial‎ roots‎ had‎ 2‎ canals‎ but‎ about‎ 13%‎ of‎ them‎ had‎ 3‎ canals.‎ This‎ was‎ similar‎ to‎ other‎ studies‎ stating‎ that‎ 2-canaled‎ roots‎ are‎ much‎ more‎ prevalent‎ (summarized‎ in‎ Table‎ 5).‎ In‎ distal‎ roots,‎ similar‎ prevalence‎ of‎ one‎ or‎ two‎ canals‎ were‎ observed‎ in‎ this‎ study‎ (with‎ very‎ few‎ cases‎ of‎ three‎ canals).‎ Such‎ a‎ high‎ frequency‎ of‎ two‎ canals‎ in‎ this‎ root‎ was‎ similar‎ to‎ studies‎ of‎ Ahmed‎ *et‎ al*‎. [21],‎ Arjmand‎ *et‎ al*‎. [10],‎ and‎ Al-Qudah‎ and‎ Awadeh‎ [14].‎ All‎ distolingual‎ roots‎ were‎ single-rooted‎ as‎ what‎ was‎ reported‎ by‎ Akhlaghi‎ *et‎ al*‎.‎ [2].‎ 

**Table‎ 1:** Distribution‎ (%)‎ of‎ canal‎ number‎ in‎ roots‎ of‎ mandibular‎ first‎ molars across mesial, distal, and distolingual roots

	**Canal** **‎** ** number**		
**Root**	**1**	**2**	**3**
**Mesial**	-	271‎ (86.9%)	41‎ (13.1%)
**Distal**	136‎ (43.6%)	165‎ (52.9%)	11‎ (3.5%)
**Distolingual**	16‎ (100%)	-	-

**Table‎ 2:** Distribution‎ of‎ canal‎ types‎ according‎ to‎ Vertucci‎ classes (I to VIII) in mesial and distal roots of mandibular first molars

		**I**	**II**	**III**	**IV** **‎**	**IV** **‎**	**V**	**VI**	**VII**	**VIII**
**Mesial root**	**N**	–	66	20	125	125	13	27	10	–
	**%**	–	21.1	6.4	40.0	40.0	4.1	8.6	3.2	–
**Distal root**	**N**	136	15	55	–	–	47	–	31	–
	**%**	43.6	4.8	17.6	–	–	15.0	–	9.9	–

**Table‎ 3:** Distribution‎ of‎ canal‎ types‎ according‎ to‎ Vertucci‎ modifications (IX to XXIII) in mesial and distal roots of mandibular first molars. Modification with all-empty cells are removed from the table

		**X**	**XII**	**XIII**	**XVI**	**XVII**	**XX**	**XXI**	**XXII**
**Mesial root**	**N**	10	9	4	–	7	13	8	–
	**%**	3.2	2.9	1.3	–	2.2	4.2	2.5	–
**Distal root**	**N**	17	–	3	4	–	–	–	4
	**%**	5.4	–	0.9	1.3	–	–	–	1.3

**Table‎ 4:** Summary‎ of‎ reports‎ on‎ number‎ of‎ roots

**Author /Country**	**Method**	**Sample size**	**Number** **‎** ** of** **‎** ** roots**		
			**1**	**2**	**3**
Kim‎ *et‎ al.* /South‎ Korea [22]	CBCT‎ (*in‎ vivo*)	1952	0.67%	73%	25%
Abella‎ *et‎ al.* [4]	review‎ of‎ literature‎ of‎ 45‎ articles	19056	-	-	14%‎ (Range‎ 0*‎ to‎ 29%)
Zhang‎ *et‎ al.*/China [23]	CBCT‎ (*in‎ vivo*)	232	0.4%	70%	29%
DePablo‎ *et‎ al.*/Spain [5]	review‎ of‎ literature‎ of‎ 41‎ articles	18787			13%‎ (Range‎ 0‎ to‎ 32%)
Demirbuga‎ *et‎ al.*/Turkey [20]	CBCT‎ (*in‎ vitro*)	1748	0.85%	95.8%	2%
Chourasia‎ *et‎ al.*/India [18]	*in* *‎* * vitro*‎ (clearing‎ technique)	150		94.6%	5.3%
Garg‎ *et‎ al.*/India [24]	*in* *‎* * vitro*‎ (periapical‎ radiography)	1054			6%
Al-Qudah,‎ Awawdeh/Jordan [14]	*in* *‎* * vitro*‎ (clearing‎ technique)	330		96%	4%
Ahmed‎ *et‎ al.*/Sudan [21]	*in* *‎* * vitro*‎ (clearing‎ technique)	100		94%	3%
Mirzaie *et‎ al.*/Iran [11]	CBCT‎ (*in‎ vivo*)	66		100%	
Ballullaya‎ *et‎ al.* [6]	review‎ of‎ literature‎ of‎ 97‎ articles				Range:‎ 3-35%
Gulabivala‎ *et‎ al.*/Myanmar [7]	*in* *‎* * vitro*‎ (clearing‎ technique)	139		90%	10%
Nur‎ *et‎ al.*/Turkey [19]	CBCT‎ (*in‎ vivo*)	966	0.3%	99.2%	0.5%
Zafar‎ *et‎ al.*/Saudi‎ Arabia [25]	CBCT‎ (*in‎ vitro*)	100		100%	
Arjmand‎ *et‎ al.*/Iran [10]	CBCT‎ (*in‎ vivo*)	121		97.5%	2.5%
Masoudi‎ *et‎ al.*/Iran [9]	CBCT‎ (*in‎ vivo*)	129		96.1%	3.9%
Akhlaghi‎ *et‎ al.*/Iran [2]	*in* *‎* * vitro*‎ (clearing‎ technique)	150		69.7%	3.3%

* *In* *‎* *studies* *‎* *from* *‎* *Iran,* *‎* *Uganda,* *‎* *Pakistan,* *‎* *Turkey,* *‎* *Kuwait* *‎* *and* *‎* *Spain,* *‎* *no* *‎* *three-root* *‎* *teeth* *‎* *had* *‎* *been* *‎* *observed*

**Table‎ 5:** Summary‎ of‎ reports‎ on‎ number‎ of‎ canals

**Author /Country**	**Method**	**Sample size**	**Number** **‎** ** of** **‎** ** canals**		
			**Overall**	**Mesial root**	**Distal root**
**Kim** **‎** ***et‎ al*‎. /South‎ Korea [22] **	**CBCT** **‎** ** (** ***in*** ***‎*** *** vivo*** **)**	1952	48.6%‎ three‎,‎ 49.2%‎ four‎	-	-
**Zhang** **‎** ***et‎ al*‎. /China [23]**	**CBCT** **‎** ** (** ***in*** ***‎*** *** vivo*** **)**	232	53%‎ three-,‎ 43%‎ four-	95%‎ two-canal	
**De Pablo** **‎** ***et‎ al*‎. /Spain [5] **	**review** **‎** ** of** **‎** ** literature** **‎** ** of** **‎** ** 41** **‎** ** articles**	18787	61%‎ three-,‎ 35.7%‎ four-	94.4%‎ two‎,‎ 2.3%‎ three‎	63%:‎ single-canal
**Demirbuga** **‎** ***et‎ al*‎. /Turkey [20]**	***in*** ***‎*** *** vitro*** **‎** ** (CBCT)**	1748	79.9%‎ three-,‎ 15.4‎ %%‎ four-	-	-
**Chourasia** **‎** ***et‎ al*‎. /India [18] **	***in*** ***‎*** *** vitro*** **‎** ** (clearing** **‎** ** technique)**	150	64%‎ three‎,‎ 36%‎ four‎		
**Al-Qudah,** **‎** ** Awawdeh/Jordan [14] **	***in*** ***‎*** *** vitro*** **‎** ** (clearing** **‎** ** technique)**	330	49%‎ three-,‎ 46%‎ four-	93%‎ two-,‎ 6%‎ three-	54%‎ single‎ 45%‎ two‎ s
**Ahmed** **‎** ***et‎ al*‎. /Sudan [21] **	***in*** ***‎*** *** vitro*** **‎** ** (clearing** **‎** ** technique)**	100		86%‎ two	59%‎ two-,‎ 38%‎ single‎,‎ 3%‎ three-
**Mirzaie** **‎** ***et‎ al*‎. /Iran [11] **	**CBCT** **‎** ** (** ***in*** ***‎*** *** vivo*** **)**	66	63%‎ three-,‎ 37%‎ four-		
**Ballullaya** **‎** ***et‎ al*‎. [6]**	**review** **‎** ** of** **‎** ** literature** **‎** ** of** **‎** ** 97** **‎** ** articles**		35%‎ four-	Three‎ canals:‎ Range‎ 0.95‎ to‎ 15%	
**Nur** **‎** ***et‎ al*‎. /Turkey [19] **	**CBCT** **‎** ** (** ***in*** ***‎*** *** vivo*** **)**	966	63%‎ three-,‎ 37%‎ four-	96.8%‎ two ,‎ 0.2%‎ three‎,‎ 3%‎ single‎	
**De Pablo** **‎** ***et‎ al*‎. /Spain [5] **	**CBCT** **‎** ** (** ***in*** ***‎*** *** vitro*** **)**	53	41.5%‎ three‎,‎ 29.4%‎ four‎,‎ 28.3%‎ five‎	96.8%‎ two ‎,‎ 0.2%‎ three‎,‎ 3%‎ single‎	
**Zafar** **‎** ***et‎ al*‎. /Saudi‎ Arabia [25] **	**CBCT** **‎** ** (** ***in*** ***‎*** *** vitro*** **)**	100		95.5%‎ two ‎	20%‎ double‎,‎ 80%‎ single‎
**Arjmand** **‎** ***et‎ al*‎. /Iran [10] **	**CBCT** **‎** ** (** ***in*** ***‎*** *** vivo*** **)**	121		92.5%‎ two ‎	58.6%‎ single‎,‎ 37.2%‎ double‎
**Masoudi** **‎** ***et‎ al*‎.‎ /Iran [9] **	**CBCT** **‎** ** (** ***in*** ***‎*** *** vivo*** **)**	129	72.1%‎ three‎,‎ 24.8%‎ four‎,‎ 1.6%‎ five‎		74.7%‎ single‎
**Akhlaghi** **‎** ***et‎ al.*/Iran [2] **	***in*** ***‎*** *** vitro*** ***‎*** ** (clearing** **‎** ** technique)**	150		100%‎ two	61.3%‎ single‎,‎ 38.7%‎ double‎

**Table‎ 6:** Summary‎ of‎ reports‎ on‎ Vertucci‎ types‎ and‎ modifications‎ (all‎ reported‎ values‎ in‎ percent)

**Author /Country**	**Method**	**Size**	**Mesial** **‎** ** root**	**Distal** **‎** ** root**	**Vertucci** **‎** ** modifications**
**Kim** **‎** ***et‎ al*‎. /South‎ Korea [22] **	**CBCT (** ***in vivo*** **)** **‎**	1952	IV‎ 71‎‎ II‎ 20‎	I‎ 66‎‎‎ II‎ 19‎‎ IV‎ 12	0.35%
**Zhang** **‎** ***et‎ al*‎. /China [23]**	**CBCT (** ***in vivo*** **)** **‎**	232	IV‎ 81‎‎ V‎ 15‎	-	-
**De Pablo** *** et*** ***‎*** *** al*** **‎** **.** ** /Spain [5]**	**review** **‎** ** of** **‎** ** literature** **‎** ** of** **‎** ** 41** **‎** ** articles**	18781	IV‎ 52.3‎‎‎ II‎ 35‎	I‎ 63‎‎ II‎ 14‎‎ IV‎ 12.4‎	
**Demirbuga** **‎** ***et‎ al*‎. /Turkey [20]**	**CBCT** **‎** ** (** ***in*** ***‎*** *** vitro*** **)**	1748	IV‎ 68 II‎ 30‎	I‎ 82‎‎ II‎ 6‎‎ IV‎ 5.6‎	
**Chourasia** **‎** ***et‎ al*‎. /India [18]**	***in*** ***‎*** *** vitro*** **‎** ** (clearing** **‎** ** technique)** **‎**	150	IV‎ 54 II‎ 36.6‎	I‎ 65.3 II‎ 20.6‎‎‎ IV‎ 9.3	
**Al-Qudah,** **‎** ** Awawdeh/Jordan [14]**	***in*** ***‎*** *** vitro*** **‎** ** (clearing** **‎** ** technique)**	330	IV‎ 53‎‎‎ II‎ 36‎	I‎ 54‎‎‎ II 17‎‎‎ V‎ 11 IV‎ 9‎	Mesial root: 5.7% Distal root: 1.7%
**Ahmed** **‎** ***et‎ al*‎. /Sudan [21]**	***in*** ***‎*** *** vitro*** **‎** ** (clearing** **‎** ** technique)**	100	IV‎ 73‎‎‎ II‎ 14‎	I‎ 38 II 28‎‎‎ V‎ 22‎‎	
**Gulabivala** **‎** ***et‎ al*‎. /Myanmar [7]**	***in*** ***‎*** *** vitro*** **‎** ** (clearing** **‎** ** technique)**	139	IV‎ 38.1‎‎‎ II 28.8‎‎‎ V‎ 6.5‎	I‎ 92.9	Mesial: 12.9% Distal: 10%
**Faraz** **‎** ***et‎ al*‎.** **[26] **	***in*** ***‎*** *** vitro*** **‎** ** (clearing** **‎** ** technique)**	123	IV‎ 70.7 II‎ 26.8‎	I‎ 65.8‎‎‎ II 14.6‎‎‎ V‎ 19.5‎	
**Nur** **‎** ***et‎ al*‎. /Turkey [19]**	**CBCT (** ***in vivo*** **)** **‎**	966	IV‎ 92‎‎‎ II‎ 5‎	I‎ 60‎‎‎ II 12 IV‎ 20‎‎‎ V‎ 7	
**Zafar** **‎** ***et‎ al*‎. /Saudi‎ Arabia [25]**	**CBCT (** ***in vitro*** **)** **‎**	100	II‎ 30 IV‎ 27.5‎‎ III‎ 20‎‎ V‎ 12.5‎	I‎ 58.6‎‎ II‎ 20.3 III 16.9‎‎	
**Arjmand** **‎** ***et‎ al*‎. /Iran [10]**	**CBCT (** ***in vivo*** **)** **‎**	121	IV‎ 65.3 II‎ 27.2‎	I‎ 47.2 III 18.9	
**Masoudi** **‎** ***et‎ al*‎. /Iran [9]**	**CBCT (** ***in vivo*** **)** **‎**	129	II‎ 62.1 IV‎ 29.5‎‎	I‎ 74.7‎‎ III 18.6‎‎ II 17.8	
**Akhlaghi** **‎** ***et‎ al*‎. /Iran [2]**	***in*** ***‎*** *** vitro*** **‎** ** (clearing** **‎** ** technique)**	150	IV‎ 55.3‎‎‎ II 41.3 VIII‎ 2.7	I‎ 61.2 II‎ 26.6 IV‎ 9.4‎	‎

The‎ most‎ common‎ types‎ of‎ canal‎ which‎ were‎ found‎ in‎ mesial‎ roots‎ of‎ this‎ study‎ were‎ Vertucci classes IV‎ (40%),‎ II‎ (21.1%)‎ and‎ VI (8.1%),‎ while‎ about‎ 16%‎ of‎ cases‎ were‎ Vertucci‎ modifications.‎ Higher‎ prevalence‎ of‎ type‎ IV‎ followed‎ by‎ type‎ II‎ was‎ seen‎ in‎ most‎ other‎ studies‎ [2, 8] except‎ those‎ reported by Zafar‎ *et‎ al*‎. [24]‎ and‎ Masoudi‎ *et‎ al*‎. [9]‎ (Table‎ 7).‎ In‎ distal‎ roots,‎ most‎ common‎ Vertucci‎ types‎ were‎ I‎ (43.6%),‎ III‎ (17.6%),‎ and‎ V‎ (15%);‎ about‎ 9%‎ of‎ cases‎ were‎ Vertucci‎ modifications‎ (Table‎ 6).‎ This‎ was‎ consistent‎ with‎ studies‎ of‎ Zafar‎ *et‎ al*‎. (37),‎ Arjamand‎ *et‎ al*‎. [10],‎ and‎ Masoudi‎ *et‎ al*‎. [9].‎ Although‎ Vertucci‎ classes‎ can‎ simplify‎ reports,‎ they are not‎ sufficient‎ to‎ cover‎ all‎ complexities‎ of‎ root‎ canal‎ structures which were‎ observed‎ in‎ this‎ study‎ and‎ few‎ others‎ that‎ have‎ evaluated‎ Vertucci‎ modifications‎ [7, 14].‎ Sometimes,‎ real‎ canals‎ are‎ much‎ more‎ irregular‎ to‎ be‎ easily‎ categorized‎ into‎ one‎ of‎ Vertucci‎ classes‎ or‎ modifications.‎ Differences‎ might‎ be‎ attributed‎ to‎ ethnicity‎.‎

## Conclusion

In this population, there were 3 to 6 canals per tooth (mostly 4 and 3 canals).‎ Males and females might be similar regarding number of roots, or number of canals in each root, or number of canals in each tooth. The most frequent Vertucci classes in mesial and distal roots were IV‎ (followed‎ by‎ II) and I‎, respectively without a significant sex dimorphism.
